# Standardisation of basal medium for reproducible culture of human annulus fibrosus and nucleus pulposus cells

**DOI:** 10.1186/s13018-018-0914-y

**Published:** 2018-08-22

**Authors:** Ann-Kathrin Schubert, Jeske J. Smink, Matthias Pumberger, Michael Putzier, Michael Sittinger, Jochen Ringe

**Affiliations:** 1 Tissue Engineering Laboratory and Berlin-Brandenburg Center for Regenerative Therapies, Charité - Universitätsmedizin Berlin, corporate member of Freie Universität Berlin, Humboldt-Universität zu Berlin and Berlin Institute of Health, Augustenburger Platz 1, Südstraße 2, 13353 Berlin, Germany; 2 CO.DON AG, Teltow, Germany; 3 Center for Musculoskeletal Surgery, Department of Orthopaedics, Charité - Universitätsmedizin Berlin, corporate member of Freie Universität Berlin, Humboldt-Universität zu Berlin and Berlin Institute of Health, Berlin, Germany

**Keywords:** Annulus fibrosus, Nucleus pulposus, Basal medium, Cell growth, Dedifferentiation, Disc marker

## Abstract

**Background:**

The lifetime prevalence of degenerative disc disease is dramatically high. Numerous investigations on disc degeneration have been performed on cells from annulus fibrosus (AF) and nucleus pulposus (NP) of the intervertebral disc (IVD) in cell culture experiments utilising a broad variety of basal culture media. Although the basal media differ in nutrient formulation, it is not known whether the choice of the basal media itself has an impact on the cell’s behaviour in vitro. In this study, we evaluated the most common media used for monolayer expansion of AF and NP cells to set standards for disc cell culture.

**Methods:**

Human AF and NP cells were isolated from cervical discs. Cells were expanded in monolayer until passage P2 using six different common culture media containing alpha-Minimal Essential Medium (alpha-MEM), Dulbecco’s Modified Eagle’s Medium (DMEM) or Ham’s F-12 medium (Ham’s F-12) as single medium or in a mixture of two media (alpha/F-12, DMEM/alpha, DMEM/F-12). Cell morphology, cell growth, glycosaminoglycan production and quantitative gene expression of cartilage- and IVD-related markers aggrecan, collagen type II, forkhead box F1 and keratin 18 were analysed. Statistical analysis was performed with two-way ANOVA testing and Bonferroni compensation.

**Results:**

AF and NP cells were expandable in all tested media. Both cell types showed similar cell morphology and characteristics of dedifferentiation known for cultured disc cells independently from the media. However, proceeding culture in Ham’s F-12 impeded cell growth of both AF and NP cells. Furthermore, the keratin 18 gene expression profile of NP cells was changed in alpha-MEM and Ham’s F-12.

**Conclusion:**

The impact of the different media itself on disc cell’s behaviour in vitro was low. However, AF and NP cells were only robust, when DMEM was used as single medium or in a mixture (DMEM/alpha, DMEM/F-12). Therefore, we recommend using these media as standard medium for disc cell culture. Our findings are valuable for the harmonisation of preclinical study results and thereby push the development of cell therapies for clinical treatment of disc degeneration.

## Background

The lifetime prevalence of degenerative disc disease (DDD) is very high. Over 90% of over age 50 show radiographic signs of DDD in magnetic resonance imaging studies [1]. Degeneration of the intervertebral disc (IVD) is a natural process occurring during ageing and is under continuous investigation [2]. The IVD is a cartilage-like tissue and consists of two compartments, the inner nucleus pulposus (NP) and the surrounding annulus fibrosus (AF). Both contain specific cells that maintain the extracellular matrix (ECM) through synthesis and degradation of ECM proteins. Cellular activity and metabolism is dependent on nutrient supply. The IVD is an avascular tissue, and thus nutrients are only supplied by the blood system of the adjacent vertebral bodies via a physical barrier, the cartilage endplate (CEP). In mature discs, the distance between an AF or NP cell and CEP is up to 6–8 mm and can only be overcome by free diffusion of fluids through the IVD tissue [3]. Although disc cells are adapted to the limited nutrient supply, an imbalance in the ECM maintenance occurs with age. The resulting impaired distribution of matrix degrading enzymes causes the DDD. To treat DDD, cell-based therapeutic approaches are of increasing clinical importance [4]. In disc cell therapy, cells are isolated from the disc and are expanded ex vivo before they are implanted into the impaired disc. Cell expansion is necessary to obtain a sufficient cell number for implantation. These strategies aim to slow down the degeneration process and are predominantly tested in preclinical studies using cell culture experiments [5].

Reviewing the literature of the two last decades, we found no common protocol available for disc cell expansion. Although cell growth and maintenance of disc cell viability is affected by nutrition [6], the cell culture medium differs with respect to serum and glucose concentration, as well as in supplementation of ascorbic acid, growth factors and non-essential amino acids. Studies even utilise a variety of basal cell culture media: alpha-Minimal Essential Medium (alpha-MEM), Dulbecco’s Modified Eagle’s Medium (DMEM) or Ham’s F-12 medium (Ham’s F-12). These synthetic culture media were originally developed for different cell types, and nutrition is changed by the medium itself. Harry Eagle found the minimal nutritional requirements to support growth and multiplication of adherent mammalian cells in vitro [7]. For more demanding cell types such as primary embryonic cells, Renato Dulbecco increased the concentration of amino acids and vitamins and added trace elements and bicarbonates resulting in DMEM [8]. DMEM has been established as universal medium for various cell types of primary source and immortalised cell lines. Another modification of Eagle’s medium, alpha-MEM, is enriched with non-essential amino acids and further vitamins for cell-lines of mouse and hamster hybrid cells [9]. Richard G. Ham, another pioneer in the field of nutritional biochemistry, defined different basal media for fibroblast cells, e.g. Ham’s F-12. Ham’s F-12 was the first medium that was used for chondrocyte expansion in cell culture [10]. In addition, alpha-MEM supports cell growth and stimulation of collagen synthesis in primary osteogenic and chondrogenic cells [11]. However, only few studies use Ham’s F-12 or alpha-MEM to culture the chondrocyte-like disc cells. For disc cell culture, DMEM is predominantly used as single medium or in a mixture with Ham’s F-12 (DMEM/F-12).

It is well known, that cell growth of primary disc cells is affected by the choice of serum and glucose concentration [12]. Furthermore, cells are altered by supplementation of growth factors or change in oxygen concentration [13, 14]. However, not much is known about the impact of the different basal media itself on human disc cells. Since nutrition is altered by the basal medium itself, we hypothesised that the choice of medium would influence the behaviour of cultured disc cells. Cells used for cell therapy could thus possibly show different characteristics, when cultured with different media. As cell expansion is the inevitable step in the manufacturing process of a cell therapeutic product, we put the focus on simple cell expansion rather than complex tissue engineering. Hence, the aim of our study was to set standards for 2D culture of human AF and NP cells with respect to the used basal medium. Therefore, we exposed human AF and NP cells to the different basal media and evaluated the cell’s response regarding cell morphology, cell growth, glycosaminoglycan (GAG) production and expression of cartilage and IVD-related genes. Although Ham’s F-12 and alpha-MEM are not commonly used as single medium for disc cell culture besides DMEM, we tested each medium separately and in a mixture of two media to see individual impacts. In order to prevent any bias by other culture conditions, we used consistent culture conditions (5% CO_2_, 95% humidity, normoxia), and all samples from all donors were exactly treated the same way regarding seeding density, time point of media exchange and subcultivation, as well as media and serum batch.

## Methods

### Tissue source and sample preparation

IVD tissue was obtained from five patients (female/male ratio 3/2) during anterior cervical fusion. All patients showed only mild radiographic degenerative changes of the IVD but failed conservative treatment (Miyazaki degeneration grade ≤ 3, Modic change ≤ 2) [15]. The mean age was 49 years (range 42–52 years).

AF and NP were macroscopically resected from the IVD tissue using lamellae appearance and tissue colour as criteria. Other tissues such as CEP were discarded. Only tissue parts with clear assignment to either AF or NP in macroscopic evaluation were used for this study. In addition, no IVD tissue with macroscopic signs of degeneration, e.g. a greyish colour or sclerotic parts, were used for this study.

### Histological analysis

To confirm correct separation of AF and NP in macroscopic preparation, we performed standard histological staining of each AF and NP sample. A representative small tissue part was embedded in Tissue-Tek® O.C.T. Compound (Sakura Finetek, Staufen, Germany), frozen in liquid nitrogen and stored at − 80 °C until sectioning. Samples were cryosectioned at a thickness of 6 μm. To show tissue morphology, sections were fixed with methanol/acetone (1:1 v/v %) and stained with Mayer’s haematoxylin (Dako, Germany) and eosin-G solution (Roth, Karlsruhe, Germany). To analyse sulphated GAGs, the sections were fixed with 4% formaldehyde (Herbeta, Germany) and stained with 1% alcian blue 8GS solution (Roth) and counterstained with nuclear fast red-aluminium sulphate solution (Roth). To demonstrate acidic GAGs, the sections were fixed with 92% ethanol and stained with 0.7% safranin O solution (Sigma-Aldrich) and subsequently counterstained with 0.2% fast green solution (Sigma-Aldrich).

### Cell isolation and cell culture

AF and NP samples were washed with phosphate-buffered saline (PBS, Biochrom, Berlin, Germany), and wet weight was determined, separately. Samples were minced, and cells were released by enzymatically treating with 3333 U/mL collagenase CLS II (Biochrom), 1 U/mL collagenase P (Sigma-Aldrich, Taufkirchen, Germany) and 333 U/mL hyaluronidase (Sigma-Aldrich) in a spinner flask under gentle stirring for 4 h at 37 °C (5% CO_2,_ 95% humidity, normoxia) [16]. After cellular release, the cell solution was put through a 100 μm nylon cell strainer (BD Falcon, Franklin Lakes, NJ, USA), centrifuged at 600×*g*, and supernatant was discarded. The viable cell number was determined using trypan blue (Sigma-Aldrich) dye exclusion. From cellular release, three NP and five AF samples of all five donors had a sufficient cell yield to perform all experiments with all media and were thus included in this study.

Cells were cultured in 12-well plates with the different media in duplicates until passage P2 under consistent culture conditions (37 °C, 5% CO_2,_ 95% humidity, normoxia). Medium was replaced every second day. For subcultivation, the cells were detached with trypsin/ethylenediaminetetraacetic acid (Biochrom) at day 6 of passage P0 and day 3 of passage P1 and P2, respectively. Seeding density was always 9000 cells/cm^2^.

### Cell culture media

AF and NP cells were cultured using six different media (all from Biochrom): (1) alpha-MEM, (2) DMEM, (3) Ham’s F-12, (4) 1:1 (v/v %) alpha-MEM and Ham’s F-12 (alpha/F-12), (5) 1:1 (v/v %) DMEM and alpha-MEM (DMEM/alpha) and (6) 1:1 (v/v %) DMEM and Ham’s F-12 (DMEM/F-12). The original formulation of alpha-MEM, DMEM and Ham’s F-12 is shown in Table 1. As the distributor only provided alpha-MEM without l-glutamine, stable l-glutamine (Biochrom) was added to alpha-MEM to achieve the glutamine concentration of the original alpha-MEM formulation (4 mM). All media were supplemented with 10% human serum (HS) (German Red Cross, Berlin, Germany) and 1% antibiotics (100 U/mL penicillin, 100 mg/mL streptomycin, Biochrom). To avoid donor variability, serum was pooled from eight donors (German Red Cross), and serum batch was not changed in this study.

**Table 1:** Original formulation of alpha-MEM, DMEM and Ham’s F-12

Substance		alpha-MEM	DMEM	Ham’s F-12
Nutrients	d-Glucose	5.6 × 10^0^	5.6 × 10^0^	1.0 × 10^1^
	Sodium pyruvate	1.0 × 10^0^	1.0 × 10^0^	1.0 × 10^0^
	Sodium bicarbonate	2.4 × 10^1^	4.4 × 10^1^	1.4 × 10^1^
	Lipoic acid	9.7 × 10^−4^	–	1.0 × 10^−3^
	Linoleic acid	–	–	3.0 × 10^−4^
	NaCl	1.2 × 10^2^	1.1 × 10^2^	1.3 × 10^2^
	KCl	5.4 × 10^0^	5.4 × 10^0^	3.0 × 10^0^
	MgSO_4_·7H_2_O	8.1 × 10^−1^	8.1 × 10^−1^	–
	MgCl_2_·6H_2_O	–	–	6.0 × 10^−1^
	Na_2_HPO_4_·H_2_O	1.1 × 10^0^	8.7 × 10^−1^	1.0 × 10^0^
	CaCl_2_·2H_2_O	1.8 × 10^0^	1.8 × 10^0^	3.0 × 10^−1^
	Fe(NO_3_)_3_·9H_2_O	–	2.5 × 10^−4^	–
	FeSO_4_·7H_2_O	–	–	3.0 × 10^−3^
	CuSO_4_·5H_2_O	–	–	1.0 × 10^−5^
	ZnSO_4_·7H_2_O	–	–	3.0 × 10^−3^
	Hypoxanthine	–	–	3.0 × 10^−2^
	Thymidine	–	–	3.0 × 10^−3^
Vitamins	Folic acid	2.3 × 10^−3^	9.1 × 10^−3^	2.9 × 10^−3^
	Vitamin B_12_	1.0 × 10^−3^	–	1.0 × 10^−3^
	Pyridoxal·HCl	4.9 × 10^−3^	2.0 × 10^−2^	–
	Pyridoxine·HCl	–	–	3.0 × 10^−4^
	Niacinamid	8.0 × 10^−3^	3.3 × 10^−2^	3.0 × 10^−4^
	d-Ca-pantothenate	2.1 × 10^−3^	8.4 × 10^−3^	1.0 × 10^−3^
	Biotin	4.1 × 10^−4^	–	3.0 × 10^−5^
	Riboflavin	2.7 × 10^−4^	1.1 × 10^−3^	1.0 × 10^−4^
	Thiamine·HCl	3.0 × 10^−3^	1.2 × 10^−2^	1.0 × 10^−3^
	Ascorbic acid	2.8 × 10^−1^	–	–
Amino acids essential	l-Isoleucine	4.0 × 10^−1^	8.0 × 10^−1^	3.0 × 10^−2^
	l-Leucine	4.0 × 10^−1^	8.0 × 10^−1^	9.9 × 10^−2^
	l-Lysine HCl	4.0 × 10^−1^	8.0 × 10^−1^	2.0 × 10^−1^
	l-Methionine	1.0 × 10^−1^	2.0 × 10^−1^	3.0 × 10^−2^
	l-Phenylalanine	2.0 × 10^−1^	4.0 × 10^−1^	3.0 × 10^−2^
	l-Threonine	4.0 × 10^−1^	8.0 × 10^−1^	1.0 × 10^−1^
	l-Tryptophan	5.0 × 10^−2^	7.8 × 10^−2^	9.8 × 10^−3^
	l-Valine	4.0 × 10^−1^	8.0 × 10^−1^	1.0 × 10^−1^
Semi-essential	l-Cysteine·H_2_O	2.2 × 10^−1^	–	2.3 × 10^−1^
	l-Tyrosine	2.0 × 10^−1^	4.0 × 10^−1^	3.0 × 10^−2^
	l-Arginine·HCl	6.0 × 10^−1^	4.0 × 10^−1^	1.0 × 10^0^
	l-Histidine·HCl·H_2_O	2.0 × 10^−1^	2.0 × 10^−1^	1.0 × 10^−1^
Non-essential	l-Alanine	2.8 × 10^−1^	–	1.0 × 10^−1^
	l-Asparagine·H_2_O	3.3 × 10^−1^	–	8.8 × 10^−2^
	l-Aspartic acid	2.3 × 10^−1^	–	1.0 × 10^−1^
	l-Glutamine	4.0 × 10^0^*	4.0 × 10^0^	1.0 × 10^0^
	l-Glutamic acid	5.1 × 10^−1^	–	1.0 × 10^−1^
	Glycine	6.7 × 10^−1^	4.0 × 10^−1^	1.0 × 10^−1^
	l-Proline	3.5 × 10^−1^	–	3.0 × 10^−1^
	l-Serine	2.4 × 10^−1^	4.0 × 10^−1^	1.0 × 10^−1^
	l-Cystine	4.2 × 10^−1^	2.0 × 10^−1^	–
Vitaminoids	Inositol	1.1 × 10^−2^	4.0 × 10^−2^	1.0 × 10^−1^
	Choline chloride	7.2 × 10^−3^	2.9 × 10^−2^	1.0 × 10^−1^
Supplements	Putrescine·2HCl	–	–	1.0 × 10^−3^
	Phenol red	2.8 × 10^−2^	4.2 × 10^−2^	2.8 × 10^−2^

Concentration is shown in mM. *As the distributor only provided alpha-MEM without l-glutamine, the user supplemented l-glutamine to achieve the original concentration in alpha-MEM

### Cell growth analysis

The cell growth of AF and NP cells was studied based on the population doubling (PD) in each passage P0, P1 and P2 and the cumulative population doubling level (cPDL) at the end of passage P2. The PD was calculated at each subcultivation with the equation PD = 3.32*(log(final cell number)−log(initial cell number)). Adding up PDs of all passages resulted in cPDL [17]. The growth rate was studied using the population doubling rate (PDR) in each passage with the equation PDR = PD/culture time [days] [18]. PD, cPDL and PDR are parameters to describe the proliferation capacity and growth rate of cell expansion, respectively.

### Glycosaminoglycan analysis

GAG deposition was analysed by alcian blue and nuclear fast red staining (see above) of the cell layer. Cells were seeded on 8-well chamber slides (BD Falcon) and cultured like the main culture. Before cells reached 100% confluency, slides were washed with PBS, fixed with acetone and stored at − 20 °C until staining.

GAG secretion was analysed with standard 1,9-dimethylmethylene blue assay using chondroitin sulphate sodium salt from shark (Sigma-Aldrich) to generate standard curves as described before [19]. Standard and samples of each donor were measured in triplicates. Culture supernatants were collected from all media exchanges and pooled within same passage. Total sample volume was determined for calculation of total amount of secreted GAGs.

### Gene expression analysis

Gene expression analysis was performed for standard cartilage marker aggrecan (ACAN), collagen type I (COL1A1), collagen type II (COL2A2) and IVD-related genes keratin 18 (KRT18) and forkhead box F1 (FOXF1). No common marker genes were available to analyse the human NP and AF phenotype, separately.

For isolation of total RNA, samples were treated with TriReagent (Sigma-Aldrich), 1-bromo-3-chloropropane extraction (Sigma-Aldrich) and purified using the RNeasy Mini Kit with on-column DNase I digestion (Qiagen, Hilden, Germany) according to the manufacturer’s instructions. The cDNA was synthesised using the Transcriptor First Strand cDNA Synthesis Kit (Roche, Mannheim, Germany) according to the manufacturer’s instructions. The gene expression analysis was performed by quantitative PCR using the primer-UPL probe system of Roche and conducted on the LightCycler® 480 (Roche). Expression of housekeeping genes ATP5F1B and RPL13A was used to normalise the individual mRNA expression level. The data are expressed as relative gene expression levels and calculated using the E-method developed by Roche (https://lifescience.roche.com). Primer and probes are shown in Table 2. The samples were measured in triplicates.

**Table 2:** Primer and probes

Gene title	Accession number^#^	Primer sequence		UPL
		forward	reverse	probe
ACAN	NM_001135.3 NM_013227.3	ctggaagtcgtggtgaaagg	tcgagggtgtagcgtgtaga	21
COL1A1	NM_000088.3	gggattccctggacctaaag	ggaacacctcgctctcca	67
COL2A1	NM_001844.4 NM_033150.2	ccctggtcttggtggaaa	cattggtccttgcattactcc	19
FOXF1	NM_001451.2	cagcctctccacgcactc	cctttcggtcacacatgct	5
KRT18	NM_000224.2 NM_199187.1	aagctggaggctgagatcg	tccaaggcatcaccaagatta	70
ATP5FB1*	NM_001686.3	agaggtcccatcaaaaccaa	tcctgctcaacactcatttcc	50
RPL13A*	NM_012423.3 NM_00127049.1	caagcggatgaacaccaac	tgtggggcagcatacctc	28

*Housekeeping genes used as reference genes in normalisation for calculation of mRNA expression level

^#^Includes all transcript variants of this gene covered by the chosen primers

### Statistical analysis

Data are expressed as mean with standard deviation. Statistical analysis between groups (media 1–6) was performed with two-way ANOVA and Bonferroni compensation (GraphPad PRISM 6.0). A *p* value of less than 0.05 was considered statistically significant.

## Results

### Characterisation of AF and NP tissue samples

**Fig. 1:**
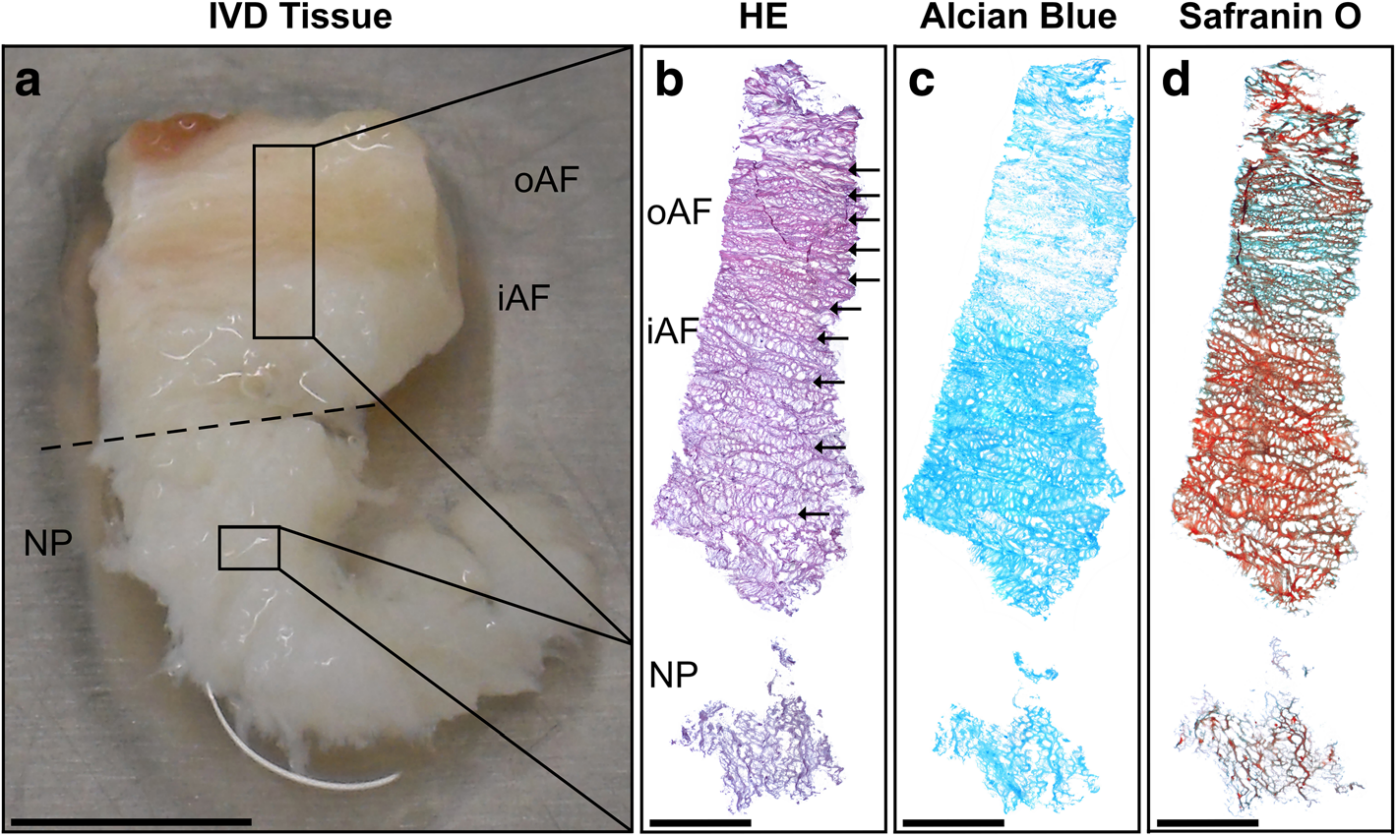
Tissue characterisation of annulus fibrosus and nucleus pulposus from human cervical intervertebral disc tissue. **a** Macroscopic separation of annulus fibrosus (AF) containing outer AF (oAF) and inner AF (iAF) from nucleus pulposus (NP) (dashed line). Histological staining for **b** tissue morphology using haematoxylin/eosin (HE) staining (lamellae indicated by arrows) and **c, d** glycosaminoglycan expression by alcian blue staining (blue) or safranin O staining (red). Exemplary shown for a 52-year-old female donor. Scale bar in **a** 1 cm and in **b**–**d** 1 mm

AF and NP tissue from cervical IVD were macroscopically distinguishable using lamellae appearance and colour as criteria (Fig. 1a). AF tissue was dense and showed its typical fibrous structure in the outer region (oAF). In the inner region of the AF (iAF), the lamellae were rather distant. NP region was white in appearance and had a soft and loose appearance. The successful separation of AF from NP was confirmed by histological evaluation. Histological staining showed the fibrillary character of the AF, whereas the NP did not contain lamellae (Fig. 1b). Furthermore, the typical zonal difference of GAG expression in oAF and iAF could be observed in alcian blue and safranin O staining (Fig. 1c and d). The GAG expression in the NP was similar to the iAF, and only appeared to be stained less strongly due to its looser tissue organisation.

After enzymatic release of the cells from the two different tissues, 860.3 ± 279.3 AF cells and 491.5 ± 120.3 NP cells per milligramme of wet tissue could be recovered on average. Hence, the cellularity in AF tissue was 1.7 as high as in NP tissue.

### Cell morphology and cell growth of primary AF and NP cells in different cell culture media

**Fig. 2:**
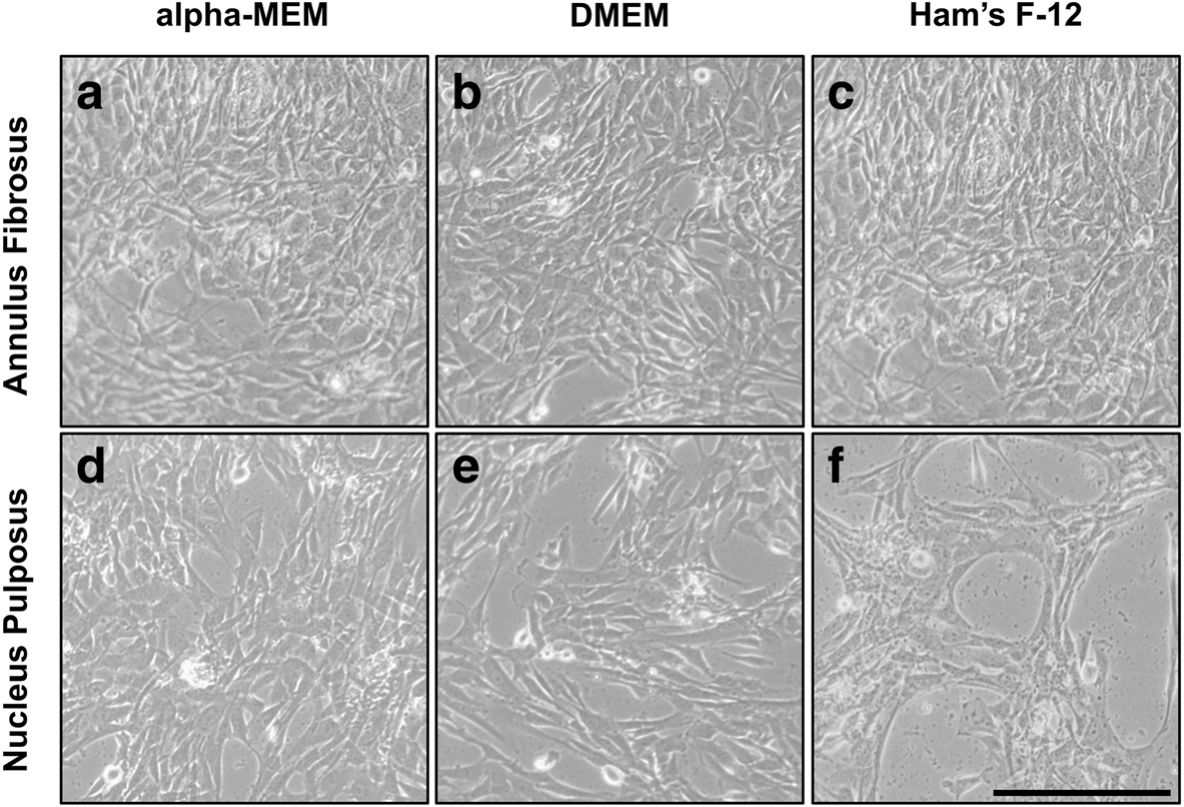
Cell morphology of disc cells exposed to different media. Exemplary shown for **a–c** annulus fibrosus and **d–f** nucleus pulposus cells (female donor, 52 years) at day 3 in passage P2 cultured in **a, d** alpha-MEM, **b, e** DMEM or **c, d** Ham’ s F-12. Scale bar 100 μm

There was no difference in cell morphology visible between cultured AF cells and NP cells. In passage P0, the cells showed isodiametric cell morphology and turned into a spindle-shaped, fibroblastic morphology through passaging. Both cell types arranged typically honeycombed at low confluency and were crowded reaching high cell density resulting in a more elongated shape (Fig. 2a–f). However, in Ham’s F-12 NP cells did not reach same confluency compared to the other media at the same culture day and detained in the honeycombed arrangement (Fig. 2f).

Nevertheless, both AF and NP cells were expandable in all tested media. Cell viability was always higher than 95%. The cell population of both AF and NP cells doubled around three times per passage (Table 3). Comparing the tested media, there was no significant difference in PD within the single passages. Nevertheless, at the end of passage P2, the cPDL of AF cells was significantly higher when cultured in alpha-MEM compared to Ham’s F-12 and alpha/F-12, as well as in DMEM/alpha compared to Ham’s F-12. In NP cells, cPDL was also significantly higher when cultured in alpha-MEM compared to Ham’s F-12 and all three media mixtures. This effect on cPDL was also significant when NP cells were cultured in DMEM compared to Ham’s F-12 or alpha/F-12.

**Table 3:** Population doubling level of disc cells cultured in different culture media

PD		alpha-MEM	DMEM	Ham’s F-12	alpha/F-12	DMEM/alpha	DMEM/F-12
AF	P0	3.5 ± 0.1	3.3 ± 0.4	2.9 ± 0.1	3.3 ± 0.1	3.5 ± 0.3	3.4 ± 0.2
	P1	3.3 ± 0.4	3.1 ± 0.8	2.6 ± 0.6	2.6 ± 0.4	3.0 ± 0.5	2.8 ± 0.6
	P2	3.3 ± 0.5	3.1 ± 0.0	3.1 ± 0.6	3.1 ± 0.6	3.4 ± 0.3	3.1 ± 0.6
	cPDL	10.2 ± 0.2 *#	9.5 ± 0.4	8.5 ± 0.8	9.0 ± 0.7	9.9 ± 0.7 *	9.3 ± 0.7
NP	P0	3.7 ± 0.4	3.3 ± 0.5	3.1 ± 0.4	2.9 ± 0.2	2.7 ± 0.7	2.8 ± 0.5
	P1	3.0 ± 0.1	2.8 ± 0.3	2.5 ± 0.3	2.5 ± 0.4	2.7 ± 0.2	2.8 ± 0.0
	P2	2.9 ± 0.2	2.9 ± 0.2	2.1 ± 0.3	2.5 ± 0.2	2.8 ± 0.3	2.8 ± 0.3
	cPDL	9.6 ± 0.3*#+§	9.1 ± 0.6*#	7.7 ± 0.5	7.8 ± 0.6	8.2 ± 0.2	8.3 ± 0.5

*PD* Population doubling in each passage P0, P1 and P2; *cPDL* cumulated PD level at the end of passage P2; *AF* annulus fibrosus; *NP* nucleus pulposus. Data is shown as mean ± SD (AF *n* = 5, NP *n* = 3).

*Significant (p value < 0.05) compared to Ham’s F-12

^#^Significant (p value < 0.05) compared to alpha/F-12

^+^Significant (p value < 0.05) compared to DMEM/alpha

^§^Significant (p value < 0.05) compared to DMEM/F-12

After seeding in passage P0, AF cells adhered to the cell culture surface within several hours, whereas NP cells took 1 to 2 days for initial attachment, independently from the medium. Furthermore, the freshly isolated cells needed some time to adapt to the cell culture conditions in vitro. This was clearly shown for both cell types in all media since the growth rate, calculated as PDR per day, was lower in passage P0 compared to the subsequent passages P1 and P2 (Table 4). Ham’s F-12 was the only medium showing both a drop in PDR of NP cells in passage P2 compared to P1 (Table 4), and a constant decrease of PDs through passaging of NP cells (Table 3). In all other media, cell growth was similar for both AF and NP cells.

**Table 4:** Population doubling rate of disc cells cultured in different culture media in each passage

PDR		alpha-MEM	DMEM	Ham’s F-12	alpha/F-12	DMEM/alpha	DMEM/F-12
AF	P0	0.6 ± 0.1	0.6 ± 0.1	0.5 ± 0.1	0.5 ± 0.1	0.6 ± 0.1	0.6 ± 0.1
	P1	1.1 ± 0.0	1.0 ± 0.2	0.8 ± 0.2	0.9 ± 0.1	1.0 ± 0.1	1.0 ± 0.1
	P2	1.1 ± 0.2	1.1 ± 0.0	1.1 ± 0.2	1.1 ± 0.2	1.2 ± 0.1	1.1 ± 0.2
NP	P0	0.6 ± 0.1	0.5 ± 0.0	0.5 ± 0.1	0.4 ± 0.1	0.4 ± 0.1	0.4 ± 0.1
	P1	1.0 ± 0.0	1.0 ± 0.1	0.9 ± 0.1	0.9 ± 0.2	1.0 ± 0.0	1.0 ± 0.0
	P2	1.0 ± 0.0	1.0 ± 0.1	0.7 ± 0.1	0.8 ± 0.1	0.9 ± 0.1	0.9 ± 0.1

*PDR* Population doubling rate (per day) in each passage P0, P1, P2; *AF* annulus fibrosus; *NP* nucleus pulposus. Data is shown as mean ± SD (AF *n* = 5, NP *n* = 3)

### Glycosaminoglycan production in AF and NP cells cultured with different media

**Fig. 3:**
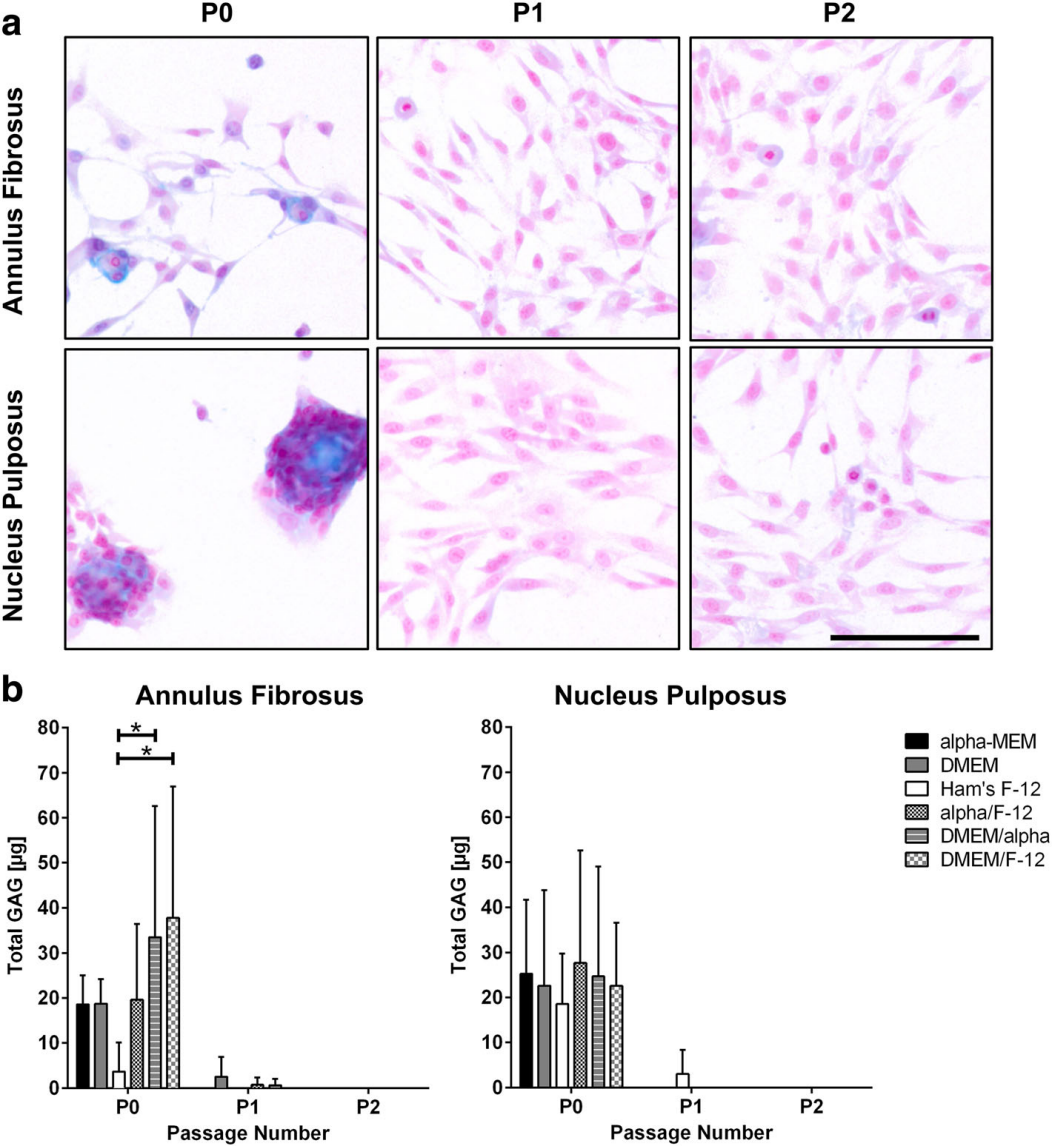
Glycosaminoglycan production through cell culture of annulus fibrosus and nucleus pulposus cells in different media. **a** Glycosaminoglycan (GAG) deposition in cells of passage P0, P1 and P2 before reaching 100% confluency (exemplary for female, 52 years, cells cultured in alpha-MEM) visualised by alcian blue staining (GAGs in blue, cell nuclei in pink). Scale bar 100 μm. **b** Total amount of secreted GAGs by cells within passage P0, P1 and P2 (shown as mean ± SD, AF *n* = 5, NP *n* = 3). *Significance (*p* value < 0.05) within same passage

Although GAGs were produced in both cell types in passage P0, GAGs could not be detected anymore in passage P1 and P2 (Fig. 3). This was independent from the tested medium and was seen in all donors. GAG deposition in AF and NP cells was only visible in passage P0 (Fig. 3a), but showed no clear trend for the different tested media. However, GAG secretion in passage P0 was significantly lower in Ham’s F-12 for AF cells, but not for NP cells (Fig. 3b).

### Gene expression profile of cartilage and IVD-related markers in AF and NP cells cultured with different media

**Fig. 4:**
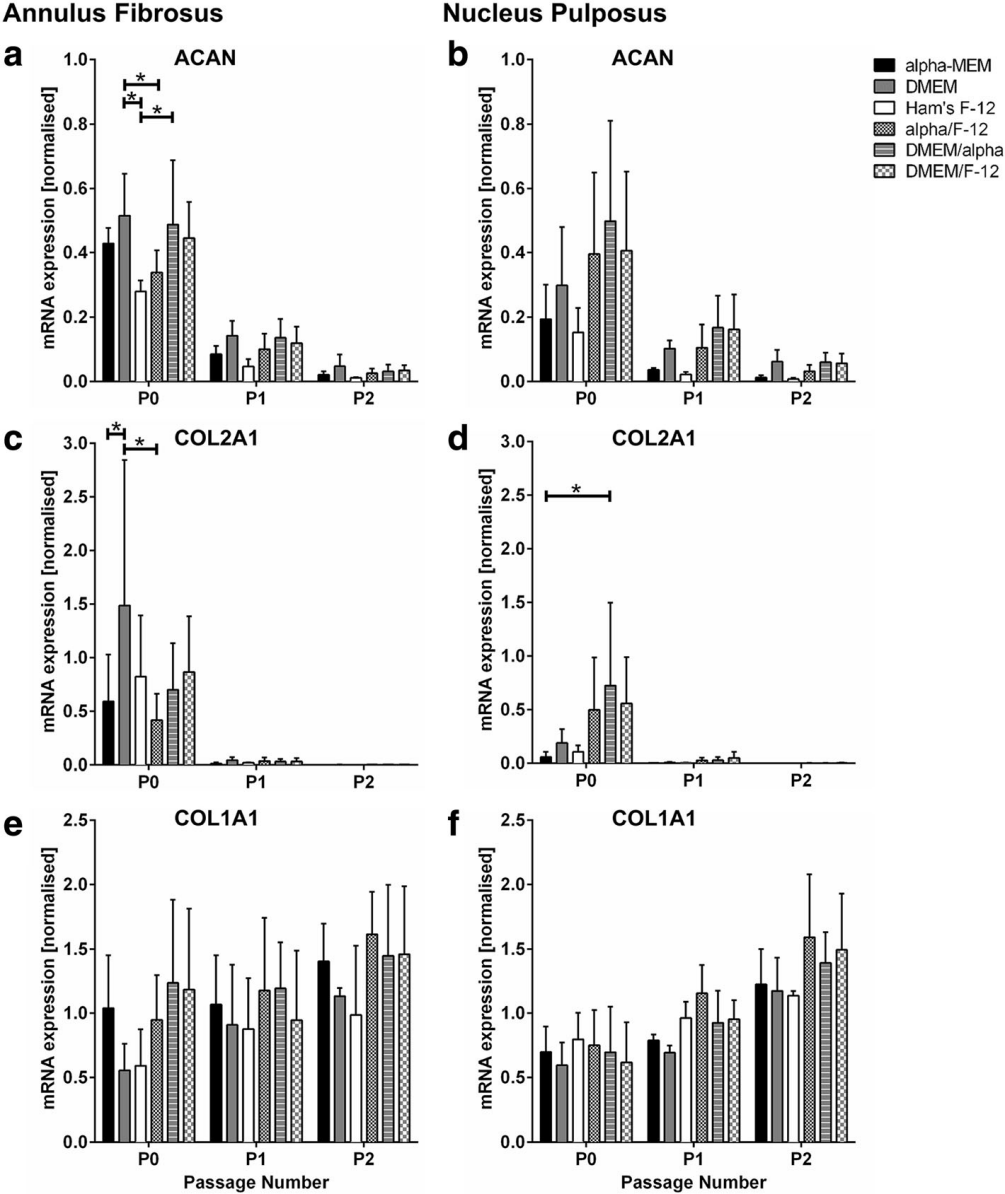
Gene expression of cartilage-related markers. **a, b** ACAN, **c, d** COL2A1 and **e, f** COL1A1 is shown for **a, c, e** annulus fibrosus and **b, d, f** nucleus pulposus cells of passage P0, P1 and P2 cultured in different media. Data is presented as mRNA expression level (normalised to reference genes ATP5FB1 and RPL13A) and shown as mean ± SD (AF *n* = 5, NP *n* = 3). *Significance (*p* value < 0.05) compared to all other media within same passage

The overall gene expression profile of the cartilage markers was independent from the tested media in both AF and NP cells. Through passaging, the standard cartilage markers ACAN and COL2A1 diminished, whereas COL1A1 slightly increased (Fig. 4). Differences between the media were only seen in ACAN and COL2A1 expression in cells of passage P0. ACAN was significantly lower expressed in AF cells cultured in Ham’s F-12 compared to DMEM and DMEM/alpha (Fig. 4a). This was also seen for NP cells but without statistical significance (Fig. 4b). COL2A1 expression was significantly lower in AF cells cultured in alpha-MEM and alpha/F-12 compared to DMEM (Fig. 4c), and in NP cells cultured in alpha-MEM compared to DMEM/alpha (Fig. 4d). COL1A1 expression showed no significant difference between the tested media in both cell types (Fig. 4e and f).

**Fig. 5:**
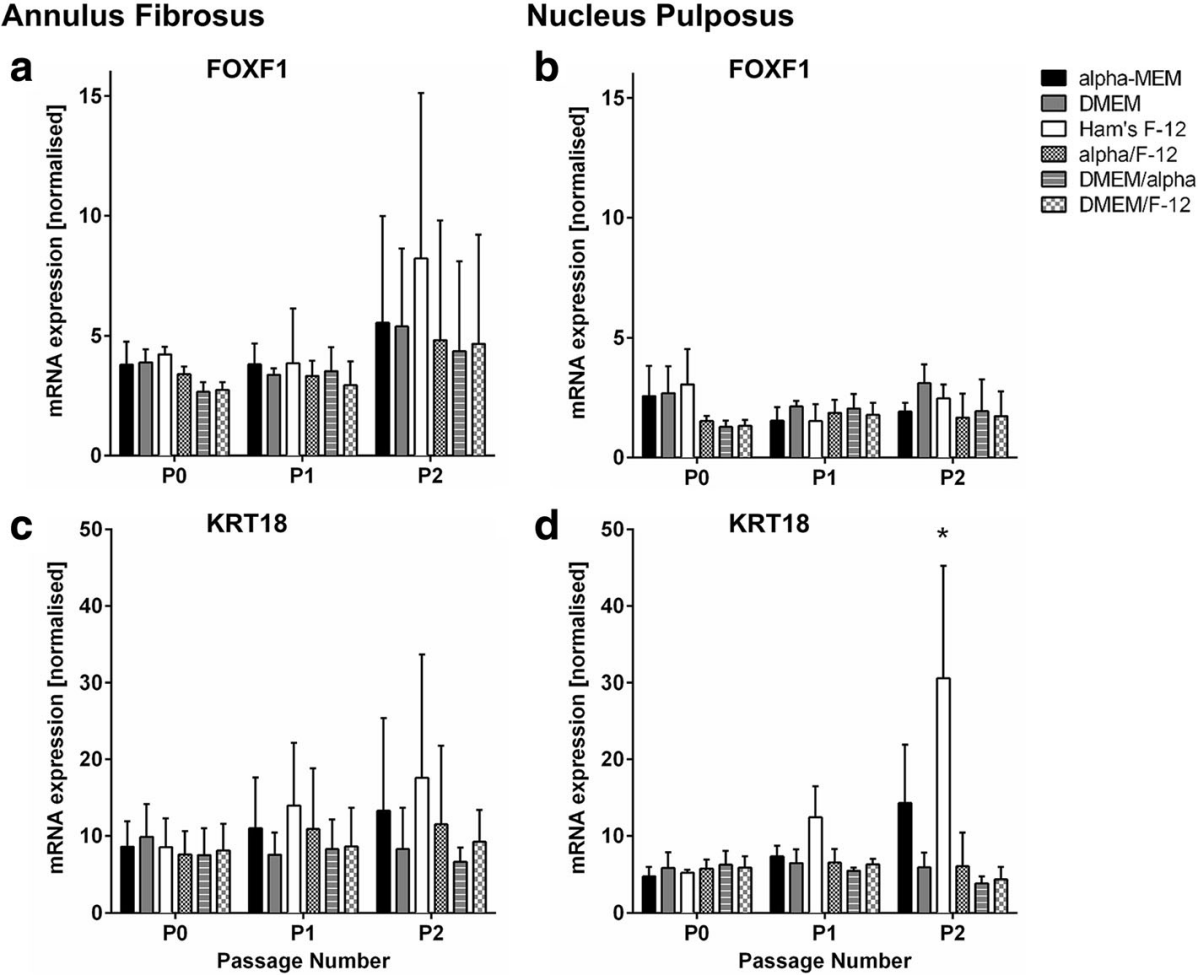
Gene expression of IVD-related markers. **a, b** FOXF1 and **c, d** KRT18 is shown for **a, c** annulus fibrosus and **b, d** nucleus pulposus cells of passage P0, P1 and P2 cultured in different media. Data is presented as mRNA expression level (normalised to reference genes ATP5FB1 and RPL13A) and shown as mean ± SD (AF *n* = 5, NP *n* = 3). *Significance (*p* value < 0.05) compared to all other media within passage

FOXF1 and KRT18 are IVD-related marker genes and thus gene expression was analysed to the see a possible impact of the tested media on the disc phenotype of cultured disc cells (Fig. 5). KRT18 and FOXF1 should have a stable expression in disc cells regardless of the passage number [20, 21]. In both AF and NP cells, FOXF1 expression was not changed by the different media (Fig. 5a and b). In AF cells, KRT18 expression was also not significantly changed through passaging or the choice of medium (Fig. 5c). Furthermore, KRT18 expression was stable in NP cells when cultured in DMEM, DMEM/alpha, DMEM/F-12 and alpha/F-12 (Fig. 5d). In contrast, KRT18 expression increased in NP cells with proceeding culture in Ham’s F-12 and alpha-MEM, and KRT18 was significantly higher expressed after two passages in Ham’s F-12 compared to all other media. Interestingly, the mRNA expression level of both FOXF1 and KRT18 was similar in AF and NP cells.

## Discussion

Only few studies are available comparing the basal media regarding cell growth, cell morphology and proteoglycan synthesis of disc cells isolated from young rats and rabbits [22, 23]. In this study, we analysed for the first time the impact of different culture media on the in vitro behaviour of primary cells isolated from AF and NP from human IVD.

The visual description of AF and NP tissue and the different AF and NP cellularity was consistent with other studies [24, 25]. In addition, initial cell attachment was delayed for cells isolated from the NP [26]. Furthermore, the histological examination of the AF and NP starting material suggested a successful separation of AF and NP during sample preparation. Hence, we exclude mixing-up of AF and NP cells, even though both cell types behaved very similar in cell culture.

As expected, both AF and NP cells showed proliferation in all tested media that are commonly used for disc cell expansion. Both AF and NP cells underwent a morphological change from an isodiametric to a spindle-shaped appearance through passaging. Furthermore, cells lost the ability to produce GAGs, as well as to express ACAN and COL2A1 after passaging, whereas COL1A1 was still present. These are typical characteristics of dedifferentiation, a process that is known for 2D culture of high specialised cells such as disc cells or cartilage cells [25, 27, 28]. The primary cells switch from their native state into a proliferative state, triggered by their attachment to the cell culture surface. Thereby, cells stop synthesising ECM molecules, e.g. GAGs and aggrecan, and switch their collagen expression from type II to type I. This was seen in all media and confirmed previous results [22]. Hence, the presence of different media did not affect dedifferentiation of cultured disc cells.

Nevertheless, our results showed that the choice of medium affected cell growth and gene expression of the IVD-related marker KRT18. The proliferation capacity of both AF and NP cells was impaired in Ham’s F-12 compared to alpha-MEM and DMEM. A lower cell growth in Ham’s F-12 compared to DMEM and a similar cell growth between alpha-MEM and DMEM was reported before for rat AF and rabbit NP cells, respectively [22, 23]. Although cell growth was highest in alpha-MEM and lowest in Ham’s F-12, both media increased the KRT18 expression in NP cells. This is a major change in the cell’s behaviour in vitro, because disc cells should typically show a stable expression of the IVD-related marker KRT18 through 2D expansion [20, 21]. As the differences between the media were only seen after proceeding culture in passage P2 but not in P0 or P1, the change in cell’s behaviour cannot be simply explained by dedifferentiation processes, but was indeed driven by the culture media and its different nutrient supply. In addition, all differences seen between the single media were compensated when the media were used in a mixture.

Comparing the media formulations, the glucose concentration is two times higher in Ham’s F-12 than in alpha-MEM and DMEM (Table 1). As glycolysis is the main energy source in disc cells [29] and glucose consumption increases with higher glucose concentration [30], we would have expected a higher performance of the cells in Ham’s F-12 compared to alpha-MEM or DMEM. However, cell growth and GAG production was lower in Ham’s F-12, and expression of cartilage-related genes was unaffected by the different media. Disc cells are used to glucose concentrations in vivo comparable to alpha-MEM, DMEM (0.56 mM) and Hams’ F-12 (1 mM). Furthermore, cell growth of human NP cells is not variant with glucose levels of either 1.8 or 2.5 mM [12]. Hence, the difference in glucose supplied by the different media was too low to have an impact that was previously described for human NP cells when cultured with either 0.5 mM or 5 mM glucose [6, 31]. This indicates that glucose was not the most important factor triggering the different performance of AF and NP cells observed in the different media.

The glutamine concentration is apparently four times higher in alpha-MEM and DMEM (4 mM) than in Ham’s F-12 (1 mM). Cell growth was higher in alpha-MEM and DMEM compared to Ham’s F-12. Other studies showed that proliferation capacity is more dependent on glutamine rather than glucose [32]. Glutamine is an alternative energy source for rapidly dividing cells that have a high demand on energy, especially when glucose level is low. So, if both glutamine and glucose are present in vitro, cells might prefer glutamine as it is faster metabolised. Nevertheless, glucose is mandatory in cell culture as the stimulatory effect of glutamine is only seen in presence of glucose [33]. The higher concentration of glucose in Ham’s F-12, however, appeared to be insufficient to compensate its lower glutamine concentration. Therefore, the continuous low glutamine level could be one reason for the lower cell growth of AF and NP cells in Ham’s F-12.

In passage P2, when decrease in cell growth was observed for NP cells in Ham’s F’12, NP cells detained in a honeycombed orientation and did not spread into free spaces between cells. A similar stratification pattern is described for immortalised AF cells when cultured in Ham’s F-12 [34]. Another study associates the cell’s behaviour with a low calcium concentration [35], comparable to the level existing in Ham’s F-12 (0.3 mM). Furthermore, calcium mediates cell attachment and cell–cell interaction through adhesion molecules, and is a major regulator in cell proliferation [36]. At a calcium concentration lower than 0.5 mM, cell proliferation is retarded [37]. Hence, AF and NP cells showed higher cell growth in alpha-MEM and DMEM compared to Ham’s F-12, because alpha-MEM and DMEM contain more calcium (1.8 mM) that is comparable to the physiological range in the blood [38]. In addition, a calcium concentration like in Ham’s F-12 was found to be more favourable for the expansion of other cell types like keratinocytes [39].

The presence of other components, for example ascorbic acid, could further have affected the cell’s behaviour in the different media. Ascorbic acid functions as a cofactor in collagen assembling and therefore facilitates ECM development. Despite the other media, alpha-MEM contains ascorbic acid (280 μM). However, both AF and NP cells were not stimulated in collagen expression on mRNA level or in GAG protein expression when cultured in alpha-MEM. By comparing with the literature, 280 μM ascorbic acid has a visible effect on GAG synthesis by disc cells [40], whereas supplementation of 1000 μM is necessary to increase collagen synthesis [41, 42]. Hence, ascorbic acid at a low concentration as used here or commonly supplemented in literature (100–280 μM) might not be relevant for disc cell culture.

We assume that low levels of other media components that are additionally present in Ham’s F-12 or rather missing in either alpha-MEM or DMEM were introduced to the cell culture as a component of the added serum. Therefore, trace elements, fatty acids, vitamins or other amino acids with slightly different concentration in the basal medium are not discussed here. In this study, we used the same serum batch constantly for all experiments. However, serum obtained from different donors contains variant concentrations of growth factors [43]. Furthermore, growth factor concentration is different in serum obtained from different origins (autologous, allogeneic, xenogeneic) and serum-replacements (like platelet rich plasma) [13, 43]. This is of importance, as the disc cell’s behaviour is altered when growth factors are supplemented to the cell culture medium [44, 45]. Hence, both the serum batch and serum origin could possibly have a greater impact on disc cells in vitro than has been seen here between the different basal media.

The overall observed difference between the tested media in passage P0 to P2 was lower than expected. It would be interesting to see the cell’s response in higher passages. However, we decided to stop cell culture in passage P2, when AF and NP cells reached a cPDL of 9.4 ± 0.8 and 8.5 ± 0.8 on average, respectively, to avoid a bias by general cell culture effects. It is known from other cartilage cells that the genetic stability is altered, when cells are cultured higher than a cPDL of 10 [46].

In agreement with other studies, the cultured AF cells were indistinguishable from NP cells regarding cell morphology and cell growth [2, 25, 47]. Furthermore and in line with literature, AF and NP cells showed similar mRNA expression level of the IVD-related marker FOXF1 and KRT18 [48, 49]. The higher KRT18 expression level in NP cells compared to AF cells was triggered by the proceeding culture in Ham’s F-12, as this was not seen for the other media. Hence, the KRT18 expression was influenced by the choice of medium and therefore nutrient supply. A previous study showed that KRT18 is down-regulated in cultured NP cells when cells are obtained from degenerated human IVD tissue [50]. An increase in KRT18 expression is only reported for NP cells, when the cell culture system is changed from 2D to 3D or throughout prolonged culture in a 3D environment [51, 52]. This further suggests that KRT18 expression in NP cells is sensitive to environmental conditions. A high ratio of aggrecan and collagen type II is another characteristic for human NP tissue [20]. Apparently, this is only true for native tissue at protein level, because we and others could not recover that ratio in primary NP cells on mRNA level [53, 54]. In addition, there was no difference between AF and NP cells in overall gene expression of ACAN, COL2A1 and COL1A1 [55]. Therefore, the common cartilage and IVD-related markers used in this study were not suitable to discriminate AF and NP cells in vitro.

Nevertheless, our results indicate that the choice of medium has an impact on the behaviour of disc cells in vitro. Furthermore, AF and NP cells were influenced differently. Ham’s F-12 impaired cell growth in NP but not in AF cells. In addition, the proceeding culture in Ham’s F-12 changed the KRT18 expression only in NP cells. Hence, it is possible that different culture media are required for AF and NP cells in order to preserve their individual cell characteristics. This is of relevance as cell-based therapeutic approaches often utilise herniated IVD tissue as starting tissue material for cell isolation [56, 57]. The herniated tissue contains a heterogeneous cell population as it originates either in the NP or AF compartment of the disc depending on the hernia location [58, 59]. Therefore, as long as no marker is available to separate human AF and NP cells, the choice of medium is important for the development of cell-therapeutic products targeted for either AF or NP repair.

## Conclusion

AF and NP cells were expandable in all tested media. The different media did not affect the typical process of dedifferentiation known for cultured disc cells. Furthermore, both cell types showed same proliferation capacity and expression of IVD-related markers when cultured in DMEM as single medium or in a mixture. In contrast, the proceeding culture in Ham’s F-12 impeded cell growth. In addition, Ham’s F-12 and alpha-MEM changed the KRT18 gene expression profile in NP cells. The observed differences between the basal media were based on its different content of glutamine and calcium rather than glucose. Therefore, the choice of medium influenced the disc cell’s behaviour in vitro as hypothesised. In conclusion, we recommend using DMEM, DMEM/alpha or DMEM/F-12 for standardised expansion of AF and NP cells in vitro. Using standardised media will generate preclinical research results with higher comparability and thus accelerate the development of cell-therapeutic approaches for disc regeneration.

## Abbreviations

2D: Two-dimensional
ACAN: Aggrecan
AF: Annulus fibrosus
alpha-MEM: Alpha-Minimal Essential Medium
ATP5F1B: ATP synthase F1 subunit beta
CEP: Cartilage endplate
COL2A1: Collagen type I
COL2A2: Collagen type II
cPDL: Cumulative population doubling level
DDD: Degenerative disc disease
DMEM: Dulbecco’s Modified Eagle’s Medium
ECM: Extracellular matrix
F-12: Ham’s F-12 medium
FOXF1: Forkhead box F1
GAG: Glycosaminoglycan
HS: Human serum
IVD: Intervertebral disc
KRT18: Keratin 18
mRNA: Messenger ribosomal nucleic acid
NP: Nucleus pulposus
PD: Population doubling
PDR: Population doubling rate
RPL13A: Ribosomal protein L13a

## References

[1] Cheung KM, Karppinen J, Chan D, Ho DW, Song YQ, Sham P, Cheah KS, Leong JC, Luk KD (2009). Prevalence and pattern of lumbar magnetic resonance imaging changes in a population study of one thousand forty-three individuals. Spine (Phila Pa 1976).

[2] Wang F, Cai F, Shi R, Wang XH, Wu XT (2016). Aging and age related stresses: a senescence mechanism of intervertebral disc degeneration. Osteoarthr Cartil.

[3] Urban JP (2002). The role of the physicochemical environment in determining disc cell behaviour. Biochem Soc Trans.

[4] Oehme D, Goldschlager T, Ghosh P, Rosenfeld JV, Jenkin G. Cell-based therapies used to treat lumbar degenerative disc disease: a systematic review of animal studies and human clinical trials. Stem Cells Int. 2015; 10.1155/2015/946031 10.1155/2015/946031 PMC4446495 26074979

[5] Mern DS, Beierfuss A, Thome C, Hegewald AA (2014). Enhancing human nucleus pulposus cells for biological treatment approaches of degenerative intervertebral disc diseases: a systematic review. J Tissue Eng Regen Med.

[6] Bibby SR, Urban JP (2004). Effect of nutrient deprivation on the viability of intervertebral disc cells. Eur. Spine J.

[7] Eagle H (1955). Nutrition needs of mammalian cells in tissue culture. Science.

[8] Dulbecco R, Freeman G (1959). Plaque production by the polyoma virus. Virology.

[9] Stanners CP, Eliceiri GL, Green H (1971). Two types of ribosome in mouse-hamster hybrid cells. Nat New Biol.

[10] Ham RG, Sattler GL (1968). Clonal growth of differentiated rabbit cartilage cells. J Cell Physiol.

[11] Pinnell SR (1985). Regulation of collagen biosynthesis by ascorbic acid: a review. Yale J Biol Med.

[12] Turner SA, Wright KT, Jones PN, Balain B, Roberts S. Temporal analyses of the response of intervertebral disc cells and mesenchymal stem cells to nutrient deprivation. Stem Cells Int. 2016; 10.1155/2016/5415901. 10.1155/2016/5415901 PMC4764757 26977156

[13] Wang SZ, Chang Q, Lu J, Wang C (2015). Growth factors and platelet-rich plasma: promising biological strategies for early intervertebral disc degeneration. Int Orthop.

[14] Gorth DJ, Lothstein KE, Chiaro JA, Farrell MJ, Dodge GR, Elliott DM, Malhotra NR, Mauck RL, Smith LJ (2015). Hypoxic regulation of functional extracellular matrix elaboration by nucleus pulposus cells in long-term agarose culture. J Orthop Res.

[15] Miyazaki M, Hong SW, Yoon SH, Morishita Y, Wang JC (2008). Reliability of a magnetic resonance imaging-based grading system for cervical intervertebral disc degeneration. J Spinal Disord Tech.

[16] Stich S, Stolk M, Girod PP, Thome C, Sittinger M, Ringe J, Seifert M, Hegewald AA (2015). Regenerative and immunogenic characteristics of cultured nucleus pulposus cells from human cervical intervertebral discs. PLoS One.

[17] Cristofalo VJ, Allen RG, Pignolo RJ, Martin BG, Beck JC (1998). Relationship between donor age and the replicative lifespan of human cells in culture: a reevaluation. Proc Natl Acad Sci U S A.

[18] Kaji K, Matsuo M (1978). Aging of chick embryo fibroblasts in vitro--I. Saturation density and population doubling rate. Exp Gerontol.

[19] Bartz C, Meixner M, Giesemann P, Roel G, Bulwin GC, Smink JJ (2016). An ex vivo human cartilage repair model to evaluate the potency of a cartilage cell transplant. J Transl Med.

[20] Risbud MV, Schoepflin ZR, Mwale F, Kandel RA, Grad S, Iatridis JC, Sakai D, Hoyland JA (2015). Defining the phenotype of young healthy nucleus pulposus cells: recommendations of the spine research interest group at the 2014 annual ORS meeting. J Orthop Res.

[21] van den Akker GG, Surtel DA, Cremers A, Rodrigues-Pinto R, Richardson SM, Hoyland JA, van Rhijn LW, Welting TJ, Voncken JW (2014). Novel immortal human cell lines reveal subpopulations in the nucleus pulposus. Arthritis Res Ther.

[22] Ichimura K, Tsuji H, Matsui H, Makiyama N (1991). Cell culture of the intervertebral disc of rats: factors influencing culture, proteoglycan, collagen, and deoxyribonucleic acid synthesis. J Spinal Disord.

[23] Rastogi A, Thakore P, Leung A, Benavides M, Machado M, Morschauser MA, Hsieh AH (2009). Environmental regulation of notochordal gene expression in nucleus pulposus cells. J Cell Physiol.

[24] Chou AI, Bansal A, Miller GJ, Nicoll SB (2006). The effect of serial monolayer passaging on the collagen expression profile of outer and inner anulus fibrosus cells. Spine (Phila Pa 1976).

[25] Kluba T, Niemeyer T, Gaissmaier C, Grunder T (2005). Human anulus fibrosis and nucleus pulposus cells of the intervertebral disc: effect of degeneration and culture system on cell phenotype. Spine (Phila Pa 1976).

[26] Horner HA, Roberts S, Bielby RC, Menage J, Evans H, Urban JP (2002). Cells from different regions of the intervertebral disc: effect of culture system on matrix expression and cell phenotype. Spine (Phila Pa 1976).

[27] von der Mark K, Gauss V, von der Mark H, Muller P (1977). Relationship between cell shape and type of collagen synthesised as chondrocytes lose their cartilage phenotype in culture. Nature.

[28] Gorensek M, Jaksimovic C, Kregar-Velikonja N, Gorensek M, Knezevic M, Jeras M, Pavlovcic V, Cor A (2004). Nucleus pulposus repair with cultured autologous elastic cartilage derived chondrocytes. Cell Mol Biol Lett.

[29] Urban JP, Winlove CP (2007). Pathophysiology of the intervertebral disc and the challenges for MRI. J Magn Reson Imaging.

[30] Bibby SR, Jones DA, Ripley RM, Urban JP (2005). Metabolism of the intervertebral disc: effects of low levels of oxygen, glucose, and pH on rates of energy metabolism of bovine nucleus pulposus cells. Spine (Phila Pa 1976).

[31] Rinkler C, Heuer F, Pedro MT, Mauer UM, Ignatius A, Neidlinger-Wilke C (2010). Influence of low glucose supply on the regulation of gene expression by nucleus pulposus cells and their responsiveness to mechanical loading. J Neurosurg Spine.

[32] Slivac I, Gaurina Srcek V, Radosevic K, Porobic I, Bilic K, Fumic K, Kniewald Z (2008). Growth characteristics of channel catfish ovary cells-influence of glucose and glutamine. Cytotechnology.

[33] Slivac I, Blajic V, Radosevic K, Kniewald Z, Gaurina Srcek V (2010). Influence of different ammonium, lactate and glutamine concentrations on CCO cell growth. Cytotechnology.

[34] van den Akker GG, Surtel DA, Cremers A, Richardson SM, Hoyland JA, van Rhijn LW, Voncken JW, Welting TJ (2016). Novel immortal cell lines support cellular heterogeneity in the human annulus fibrosus. PLoS One.

[35] Hennings H, Michael D, Cheng C, Steinert P, Holbrook K, Yuspa SH (1980). Calcium regulation of growth and differentiation of mouse epidermal cells in culture. Cell.

[36] Ko KS, Arora PD, Bhide V, Chen A, McCulloch CA (2001). Cell-cell adhesion in human fibroblasts requires calcium signaling. J Cell Sci.

[37] Boynton AL, Whitfield JF, Isaacs RJ, Morton HJ (1974). Control of 3T3 cell proliferation by calcium. In Vitro.

[38] Goldstein DA. Serum calcium. In: Walker HK, Hall WD, Hurst JW, editors. Clinical Methods: The History, Physical, and Laboratory Examinations. 3rd ed. Boston: Butterworths; 1990. Available from: https://www.ncbi.nlm.nih.gov/books/NBK201/. 21250045

[39] Dahm AM, de Bruin A, Linat A, von Tscharner C, Wyder M, Suter MM (2002). Cultivation and characterisation of primary and subcultured equine keratinocytes. Equine Vet J.

[40] Lee YJ, Kong MH, Song KY, Lee KH, Heo SH (2008). The relation between Sox9, TGF-beta1, and proteoglycan in human intervertebral disc cells. J Korean Neurosurg Soc.

[41] Flagler DJ, Huang CY, Yuan TY, Lu Z, Cheung HS, Intracellular Flow GWY (2009). Cytometric measurement of extracellular matrix components in porcine intervertebral disc cells. Cell Mol Bioeng.

[42] Kim MH (2006). Effect of L-ascorbic acid on collagen synthesis in 3T6 fibroblasts and primary cultured cells of chondrocytes. J Korean Soc Food Sci Nutr.

[43] Tallheden T, van der Lee J, Brantsing C, Mansson JE, Sjogren-Jansson E, Lindahl A (2005). Human serum for culture of articular chondrocytes. Cell Transplant.

[44] Gruber HE, Fisher EC, Desai B, Stasky AA, Hoelscher G, Hanley EN (1997). Human intervertebral disc cells from the annulus: three-dimensional culture in agarose or alginate and responsiveness to TGF-beta1. Exp Cell Res.

[45] Hegewald AA, Cluzel J, Kruger JP, Endres M, Kaps C, Thome C (2014). Effects of initial boost with TGF-beta 1 and grade of intervertebral disc degeneration on 3D culture of human annulus fibrosus cells. J Orthop Surg Res.

[46] Wallenborn M, Petters O, Rudolf D, Hantmann H, Richter M, Ahnert P, Rohani L, Smink JJ, Bulwin GC, Krupp W (2018). Comprehensive high-resolution genomic profiling and cytogenetics of human chondrocyte cultures by GTG-banding, locus-specific FISH, SKY and SNP array. Eur Cell Mater.

[47] Chen YF, Zhang YZ, Zhang WL, Luan GN, Liu ZH, Gao Y, Wan ZY, Sun Z, Zhu S, Samartzis D (2013). Insights into the hallmarks of human nucleus pulposus cells with particular reference to cell viability, phagocytic potential and long process formation. Int J Med Sci.

[48] van den Akker GGH, Koenders MI, van de Loo FAJ, van Lent P, Blaney Davidson E, van der Kraan PM (2017). Transcriptional profiling distinguishes inner and outer annulus fibrosus from nucleus pulposus in the bovine intervertebral disc. Eur Spine J.

[49] Rutges J, Creemers LB, Dhert W, Milz S, Sakai D, Mochida J, Alini M, Grad S (2010). Variations in gene and protein expression in human nucleus pulposus in comparison with annulus fibrosus and cartilage cells: potential associations with aging and degeneration. Osteoarthr Cartil.

[50] Lv FJ, Peng Y, Lim FL, Sun Y, Lv M, Zhou L, Wang H, Zheng Z, Cheung KM, Leung VY (2016). Matrix metalloproteinase 12 is an indicator of intervertebral disc degeneration co-expressed with fibrotic markers. Osteoarthr Cartil.

[51] Sun Y, Lv M, Zhou L, Tam V, Lv F, Chan D, Wang H, Zheng Z, Cheung KM, Leung VY (2015). Enrichment of committed human nucleus pulposus cells expressing chondroitin sulfate proteoglycans under alginate encapsulation. Osteoarthr Cartil.

[52] Wan S, Borland S, Richardson SM, Merry CL, Saiani A, Gough JE (2016). Self-assembling peptide hydrogel for intervertebral disc tissue engineering. Acta Biomater.

[53] Park JY, Kuh SU, Park HS, Kim KS (2011). Comparative expression of matrix-associated genes and inflammatory cytokines-associated genes according to disc degeneration: analysis of living human nucleus pulposus. J Spinal Disord Tech.

[54] Choi EH, Park H, Park KS, Park KS, Kim BS, Han IB, Shin DA, Lee SH (2011). Effect of nucleus pulposus cells having different phenotypes on chondrogenic differentiation of adipose-derived stromal cells in a coculture system using porous membranes. Tissue Eng Part A.

[55] Sive JI, Baird P, Jeziorsk M, Watkins A, Hoyland JA, Freemont AJ (2002). Expression of chondrocyte markers by cells of normal and degenerate intervertebral discs. Mol Pathol.

[56] Meisel HJ, Siodla V, Ganey T, Minkus Y, Hutton WC, Alasevic OJ (2007). Clinical experience in cell-based therapeutics: disc chondrocyte transplantation a treatment for degenerated or damaged intervertebral disc. Biomol Eng.

[57] Tschugg A, Diepers M, Simone S, Michnacs F, Quirbach S, Strowitzki M, Meisel HJ, Thome C (2017). A prospective randomized multicenter phase I/II clinical trial to evaluate safety and efficacy of NOVOCART disk plus autologous disk chondrocyte transplantation in the treatment of nucleotomized and degenerative lumbar disks to avoid secondary disease: safety results of phase I-a short report. Neurosurg Rev.

[58] Moore RJ, Vernon-Roberts B, Fraser RD, Osti OL, Schembri M (1996). The origin and fate of herniated lumbar intervertebral disc tissue. Spine (Phila Pa 1976).

[59] Rajasekaran S, Babu JN, Arun R, Armstrong BR, Shetty AP, Murugan S (2004). ISSLS prize winner: a study of diffusion in human lumbar discs: a serial magnetic resonance imaging study documenting the influence of the endplate on diffusion in normal and degenerate discs. Spine (Phila Pa 1976).

